# Can Common Functional Gene Variants Affect Visual Discrimination in Metacontrast Masking?

**DOI:** 10.1371/journal.pone.0055287

**Published:** 2013-01-24

**Authors:** Margus Maksimov, Mariliis Vaht, Jaanus Harro, Talis Bachmann

**Affiliations:** University of Tartu, Tartu, Estonia; University of Leicester, United Kingdom

## Abstract

Mechanisms of visual perception should be robustly fast and provide veridical information about environmental objects in order to facilitate survival and successful coping. Because species-specific brain mechanisms for fast vision must have evolved under heavy pressure for efficiency, it has been held that different human individuals see the physical world in the same way and produce psychophysical functions of visual discrimination that are qualitatively the same. For many years, this assumption has been implicitly accepted in vision research studying extremely fast, basic visual processes, including studies of visual masking. However, in recent studies of metacontrast masking surprisingly robust individual differences in the qualitative aspects of subjects' performance have been found. As the basic species-specific visual functions very likely are based on universal brain mechanisms of vision, these differences probably are the outcome of variability in ontogenetic development (i.e., formation of idiosyncrasic skills of perception). Such developmental differences can be brought about by variants of genes that are differentially expressed in the course of CNS development. The objective of this study was to assess whether visual discrimination in metacontrast masking is related to three widely studied genetic polymorphisms implicated in brain function and used here as independent variables. The findings suggest no main effects of BDNF Val66Met, NRG1/rs6994992, or 5-HTTLPR polymorphisms on metacontrast performance, but several notable interactions of genetic variables with gender, stage of the sequence of experimental trials, perceptual strategies, and target/mask shape congruence were found. Thus, basic behavioral functions of fast vision may be influenced by common genetic variability. Also, when left uncontrolled, genetic factors may seriously confound variables in vision research using masking, obscure clear theoretical interpretation, lead to unexplicable inter-regional differences and create problems of replicability of formerly successful experiments.

## Introduction

**Figure 1:**
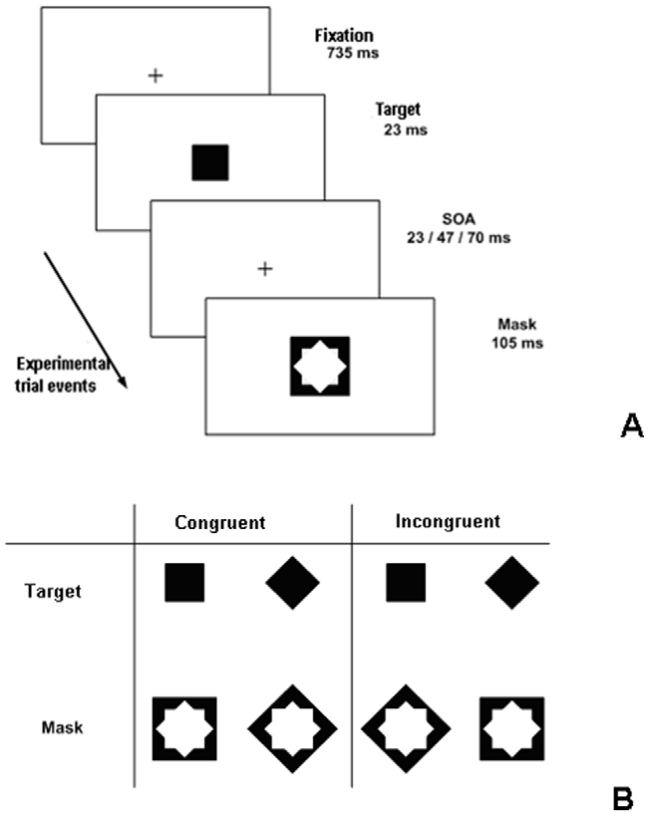
Examples of targets and masks and events in an experimental trial. Illustration of the typical sequence of events in a trial of metacontrast masking (A) and examples of the stimuli (B). Targets precede masks by SOAs of 23, 47, or 70 ms and target/mask shapes can be either congruent or incongruent in single trials.

Visual masking refers to impairment of perception of a target stimulus as a result of presentation of the masking stimulus in close spatiotemporal proximity to a target [1], [2]. In the metacontrast variety of masking a briefly presented target is followed in time by a mask so that the two stimuli do not overlap spatially, but are closely adjacent (see Figure 1A). Metacontrast has been a useful tool in studying visual functions when factors involved in fine-scale spatial and temporal discrimination are of interest, when it is necessary to present some information unconsciously to study its effects (e.g., in masked priming), and when mechanisms of perceptual consciousness are to be studied [1], [3], [4], [5]. Traditionally, metacontrast research has been nomothetic in its approach, aiming at specifying universal regularities and limitations of visual information processing by the brain. The underlying implicit assumption has been that the basic visual processes involved in fast perception of simple visul stimuli are qualitatively the same between different individuals, being founded on species-specific brain mechanisms universally shared in the population. However, a recent twist in metacontrast studies has added some idiographic flavour – substantial and stable individual differences in the types of metacontrast functions have been found [6], [7], [8], [9]. Both with German [6], [8] and Estonian [7] populations of subjects the same task of discrimination of metacontrast-masked targets in invariant experimental conditions has produced striking individual variability in the qualitative types of masking. Some of the subjects produced type-A masking (monotonic increase in target discriminability with increase in stimulus onset asynchrony, SOA, between target and mask), while other subjects showed type-B masking (nonmonotonic function where the shortest SOAs lead to better performance than intermediate SOAs). Individual differences in behavior may be caused by genetic factors leading to phenotypal variability and by learning in the course of ontogenetic development and aquisition of cognitive-perceptual skills. However, no data is available on the possible contribution of the genetic factors in the expression of the skills related to visual masking. Consequently, we carried out an exploratory study to see whether metacontrast effects may depend on genetic polymorphisms known to be related to development of the brain and brain function. We conjectured that if the answer will be affirmative and some of the typical variables involved in metacontrast masking will show dependence of their effects on genetic variability, the direction for more rigorous follow-up studies targeted specifically at investigating the effects of these genes (specifically on the selected variables) will be mapped.

From recent experiments [7], [8] we know that the type of masking function depends on whether target and mask stimuli shapes are congruent or incongruent (see Figure 1B for examples of congruent and incongruent pairings of target/mask shapes, e.g., a square or diamond). Target/mask *congruence* influences the criterion contents subjects use in evaluating targets vis-à-vis the mask [7], [8]. This is another factor of interest. Criterion contents are a typical source of bias effect in perceptual reports and substantially predetermine whether type-A or type-B functions appear [1], [8]. It is known that criterion effects largely depend on an interaction of frontal and posterior cortical processes and therefore data on genetic polymorphisms' effects on differences between relative levels of hemodynamic activity and inter-regional connectivity involving DLPFC, medial PFC, orbitofrontal cortex on the one hand and inferior temporal cortex, lateral temporal cortex and (para)hippocampal areas on the other hand [10], [11], [12] suggest that respective polymorphisms may ineract with behavioural effects of masking.

We examined the metacontrast effects in subjects genotyped for three functional polymorphisms known to affect brain structure: (1) serotonin (5-HT) transporter gene ins/del polymorphism (5-HTTLPR) using biallelic classification, (2) brain derived neurotrophic factor (BDNF) Val66Met polymorphism, and (3) neuregulin 1 (NRG1) a promoter polymorphism SNP8NRG243177 (rs6994992).

Carriers of the 5-HTTLPR short allele display increased amygdala reactivity to fearful stimuli [13], reduced hippocampal [14] and gray matter volume in perigenual cingulate and amygdala [15] and enhanced functional coupling between the amygdala and the ventromedial prefrontal cortex [16].

In the case of BDNF Val66Met polymorphism, Met allele carriers are characterized by deficits in neural plasticity and decrease of the neocortical gray matter volume [17]; as Val66Met polymorphism considerably disturbs BDNF secretion for hippocampal neurones it follows that the principal hippocampal functions suffer as well [18].

NRG1 rs6994992 minor (T) allele has been associated with decreases in white matter density in the right anterior internal capsule [19], reduced white matter integrity in the left anterior thalamic radiation [20], decreased grey matter volume in several frontal gyri, as well as decreased white matter volume in the regions of the genu and body of the corpus callosum [21]. No data is available on the effcts of the above listed three gene polymorphisms on basic visual functions as they emerge in visual metacontrast masking.

## Materials and Methods

### Participants

The opportunistic sample consisted in 57 Estonian subjects (age range 18–45, M = 26.7, 29 female and 28 male subjects) with normal or corrected-to-normal vision. The data from subjects whose correct response rate did not exceed the random correct quessing level in a particular condition were not used in the data analysis. To participate, subjects had to be in good health, having no tiredness and having consumed no alcohol or other substances potentially confounding the experimental results.

### Ethics statement

Experimental procedures were approved by the Research Ethics Committee of the University of Tartu under the general project “Comparative analysis of the effects of different attentional perception paradigms by the methods of transcranial magnetic stimulation and EEG.” Subjects recruited to the study consented to an additional procedure of giving saliva samples for genotyping in a subset of experiments without TMS/EEG provided their written informed consent including the following statements: “... NB! Participation in the study is voluntary and you may at any moment to withdraw from it and/or discontinue the experiment. Data gathered during this research will not be made available to third parties in the format allowing to identify the participating person unless that person gives his/her consent to do so.” All subjects who signed the informed consent form consented to giving samples of saliva for genotyping. (One subject from the initial sample of 58 withdrew from the study upon hearing that a sample of saliva for genotyping may be requested.) Signed forms of consent are kept by the first author so as to guarantee confidentiality and all data from genotyping and metacontrast results is used in an anonymous format.

### Behavioral experiment

#### Stimuli and procedure

Two types of stimuli were used – targets and masks. Targets were solid black squares and diamonds both subtending 1.5 degrees of visual angle in diameter; masks were prepared as black squares or diamonds subtending 2.6 degrees, with inner empty spaces shaped so as to allow the 1.5 degree targets fit in snugly within mask contours (see Figure 1B for examples of targets and masks and possible pairings between a target and a mask). Stimuli were presented on light background (72 cd/m^2^). In each trial shapes of a target and a mask were either congruent or incongruent. The spatial separation between targets' outer edges and masks' inner edges was 0.02 degrees of visual angle.

Each trial consisted in presentation of a fixation cross (735 ms), a pseudo-randomly chosen target (23 ms), followed either by a mask or by a fixation field which in turn was followed by a pseudo-randomly chosen mask (105 ms). Thus, SOAs between the target and mask varied between 23, 47, and 70 ms. (See Figure 1A for an example of the events in a trial.) (The constraint was that, overall, in each condition there will be half of the trials with congruent target/mask pairings and half of the trials with incongruent target/mask pairings. The task of subjects was to discriminate the target shapes (forced choice responding; chance level of correct responses equal to 0.5). The viewing distance to the computer monitor was 60 cm. Stimuli were displayed in the center of the screen (Eizo Flex Scan T550, refresh rate 85 Hz); after each trial subjects used a mouse to click at the icon corresponding to the target alternative they perceived. When the response was incorrect, a 1000-Hz sound was presented. Before the main experiment, subjects were able to practice the task and ask necessary questions from experimenters concerning the task and procedure. Upon completing the target discrimination task subjects were asked to describe what were, according to their introspective experience, the visual perceptible characteristics and attributes of the stimuli on which they founded their responses. Thus, in addition to measuring the rate of correct responding a qualitative analysis was carried out. Subjects answered four questions: (i) what visual characteristics/cues subjects used when discriminating the target, (ii) did the strategy for the responses change during the experiment, (iii) what was the focus of attention and its trajectory (if relevant), (iv) if there was a change in strategy and/or focus, how might this have influenced the results?

### Genotyping

After subjects completed the qualitative questionnaire, a 2 ml sample of saliva was taken from each subject in order to extract DNA; a stabilizing solution was added to the sample to guarantee its preservation and later use. For collecting the samples the SaliGene® Saliva Collection Set (manufactured by Invitek GmbH) was used, handled according to the instructions of the manufacturer. To extract DNA from the sampleQiagen QIAamp Mid set was used. Genotyping of the acquired DNA was performed based on TaqMan® Pre-Designed SNP Genotyping Assays technology (using the Applied Biosystems equipment ViiA™ 7 Real-Time PCR). The procedure was carried out in the Institute of Technology of the University of Tartu.

As a result of genotyping, the following sizes of subjects' sub-samples were found: with regard to BDNF Val66Met polymorphisms there were 32 Val/Val homozygotes and 20 Met allele carriers; with regard to NRG1/rs6994992 polymorphisms there were 18 C/C homozygotes and 34 T allele carriers; with regard to 5HTTLPR polymorphisms there were 24 L/L carriers and 28 S allele carriers (24 L/S heterozygotes and 4 S/S homozygotes).

## Results

**Figure 2:**
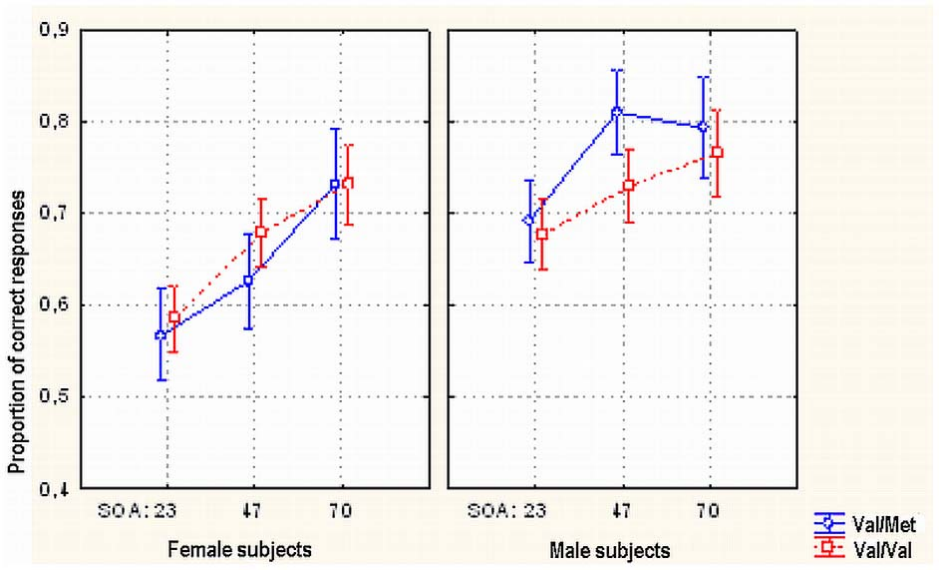
Effects of gender, SOA and BDNF Val66Met gene polymorphisms on masking. Proportion of correct target discrimination responses as a function of subjects' gender, SOA between target and mask and gene polymorphisms of the subjects (Val/Val vs Val/Met). Met allele carrying males show relatively higher correct perception rate with intermediate SOA.

**Figure 3:**
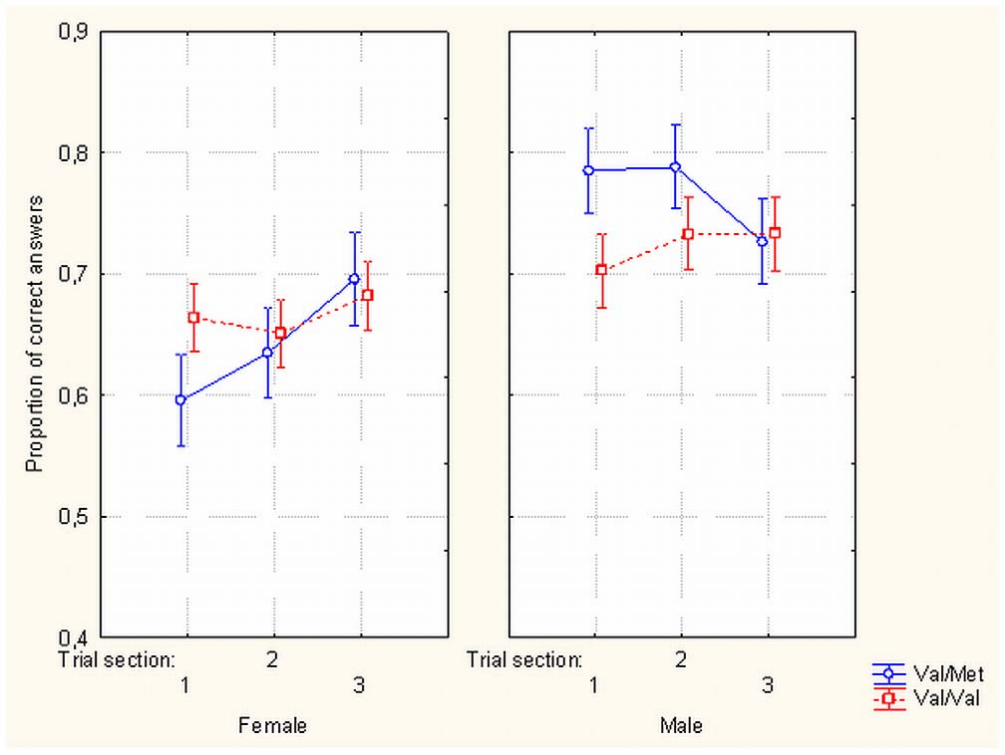
Effects of gender, stage of experiment and BDNF Val66Met gene polymorphisms on masking. Proportion of correct target discrimination responses as a function of subjects' gender, stage of the experiment (first, second or third section in a tripartite division of the successive trials) and gene polymorphisms of the subjects (Val/Val vs Val/Met). Met allele carrying males show relatively higher correct perception rate early in the experiment.

**Figure 4:**
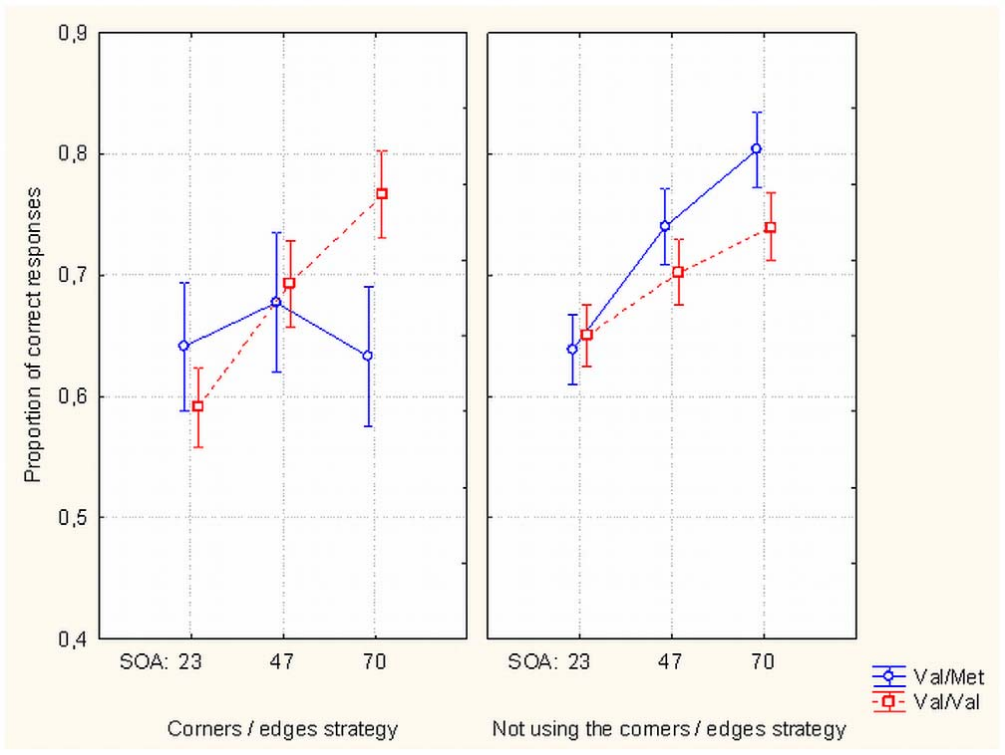
Effects of SOA, perceptual strategy and BDNF Val66Met gene polymorphisms on masking. Proportion of correct target discrimination responses as a function of SOA between target and mask, perceptual strategy used by subjects (concentrate on corners/edges of targets near the mask inner contours or not using this strategy) and gene polymorphisms of the subjects (Val/Val vs Val/Met). Met allele carrying subjects who do not use the corners/edges perceptual strategy show relatively higher correct perception rate with longest SOA.

We found no main effect of BDNF polymorphisms on correct discrimination of targets in metacontrast (*F*_(2, 51)_  = .234, *p* = .792). There were several significant interactions involving BDNF endophenotypes. (i) Female Val/Val homozygotes did not have a lower rate of correct discrimination than Met allele carriers, but male Val/Val homozygotes had – an interaction (*F*_(1, 53)_  = 4.678, *p*<.035) which substantially originates from intermediate SOA conditions where the proportion of correct responses in males was significantly higher (Tukey HSD post hoc test *p* <.002; *t*_(20)_  = −4.087, *p*<.0006, Cohen's d = 1.88). In Val/Val homozygotes no analogous gender difference was found (Tukey HSD post hoc test *p* = .520, *t*_(33)_  = −1.330, *p* = .193) (See Figure 2). A similar genotype x gender interaction was found for the parahippocampal, right DLPFC, various left temporal and right caudate rCBF levels of female and male Val/Val and Val/Met carriers [10]: Val/Val females had higher activity than Val/Val males whereas Val/Met males had higher activity than Val/Met females.

(ii) A three-way interaction between gender, genotype and stage of the experiment (*F*_(2, 106)_  = 4.566, *p*<.013) showed different dynamics of metacontrast discrimination for different genotypes in males and females. In the Val/Met group in the first stage of the experiment, females produced significantly fewer correct answers than males (t_(20)_  = −3.5, *p*<.002, Cohen's d = 1.67). After Bonferroni correction the effect remained at a significant level (df 89.9, p<.026). Differences in the proportion of correct answers between gender groups disappeared in the end sections of the experiment (t_(20)_  = −0.498, *p* = .624). (See Figure 3).

(iii) Val/Val homozygotes performed similarly regardless of the perceptual strategy, but Met allele carriers' performance dropped with a longer SOA when they used the strategy of paying attention to local corners/edges of the target situated within the inner mask contours (F_(2, 106)_  = 3.213, *p*<.044) (see Figure 4). At SOA  = 70 ms Met allele carriers who used the corners/edges strategy performed at a significantly lower level than Met allele carriers who did not use this strategy (*t*_(20)_  = 2.328, *p*<.031). Whether this may be related to poorer recognition memory of Met allele carriers [18] or some other influences on cognitive processes stemming from lower activity of Met allele remains to be explored in future. For example, Met allele carriers were found to have unstable iconic memory while in Val homozygotes iconic memory duration was longer [22]. Drop of discrimination with longer SOA in Met allele carriers in our study may be interpreted as a result of decreased visual persistence of information about target corners/edges, which has its strongest effect in the conditions where target-mask spatiotemporal integration is less pronounced (i.e., with longer SOA).

**Figure 5:**
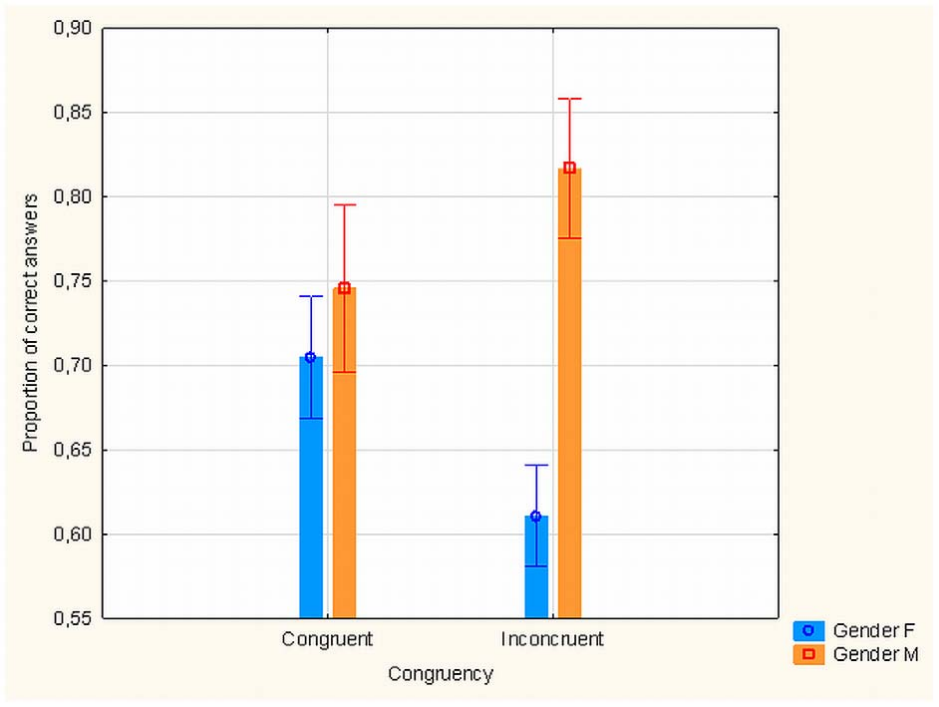
Effects of gender, target/mask congruence and NRG1/rs6994992 gene polymorphisms on masking. Proportion of correct target discrimination trials as a function of subjects' gender and target/mask shape congruence in the sub-sample of subjects, T allele carriers of the NRG1/rs6994992 gene polymorphism. When target and mask stimuli shapes were incongruent, males' level of metacontrast discrimination was higher than females' level of metacontrast discrimination while gender had no significant effect when target and mask shapes were congruent.

There was neither a main effect of NRG1/rs6994992 polymorphism or gender on metacontrast nor any gender and polymorphism interaction. However, a four-way significant interaction between gender, polymorphism, SOA and target/mask shape congruence (*F*_(4, 102)_  = 4.114, *p*<.004) suggested a need for separate analysis carried out separately for different gender groups. (To remind the non-specialist reader – SOA specifies the time interval between target and mask onsets and congruence specifies whether target and mask shapes were congruent or incongruent; see Figure 1B.) In females, the interaction between NRG1/rs6994992, SOA and target/mask shape congruence was not significant, but in males it was (*F*_(4, 50)_  = 3.536, *p*<.013). How the similarity of target and mask shapes relates to target/mask relative timing appears important for male subjects. In the group of T allele carriers when target and mask shapes were incongruent, males' level of metacontrast discrimination was higher than females' level of metacontrast discrimination (*M*_(SD = 0.144)_  = 0.817 vs *M*_(SD = 0.124)_  = 0.611, respectively; *t*_(27)_  = −4.034, *p*<.0004). Gender of T allele carriers had no significant effect when target and mask shapes were congruent (see Figure 5). This raises an important question: in the metacontrast experiments where target/mask shape congruence is varied [6], [7], [8] and appears to have an effect on the type of masking (e.g., type-A, monotonic functions of SOA and type-B, non-monotonic functions) it should be important to know the genotype of the subjects. When subjects groups include relatively more male participants carrying T allele then a higher level of metacontrast discrimination with incongruent target/mask pairings may be more likely. At first this may seem unexpected because earlier data has shown structural and functional underdevelopment in the brains of T allele carriers suggesting suboptimal frontal-cortex functioning in this group [21], [23]. Paradoxically, this kind of a relative lack of cognitive flexibility in a perceptual task which seems to be characteristic to T allele carriers may lead to a better task performance in specific circumstances.

**Figure 6:**
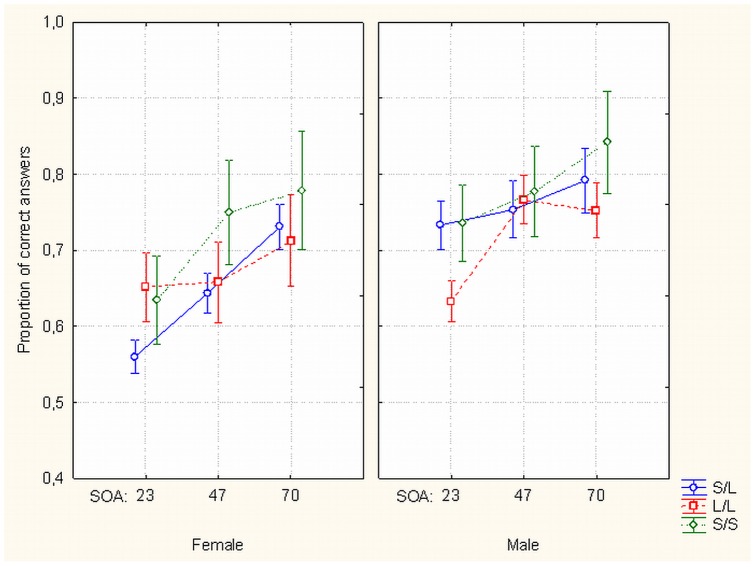
Effects of gender, SOA and 5-HTTLPR gene polymorphisms on masking. Proportion of correct target discrimination responses as a function of subjects' gender, SOA between target and mask and gene polymorphisms of the subjects (5-HTTLPR, L/L homozygotes vs S/L heterozygotes). With the shortest SOA female L/L homozygotes had higher rate of correct discrimination than S/L heterozygotes while in males this pattern of results was reversed.

There was no main effect of 5-HTTLPR polymorphism on metacontrast masking (*F*_(2, 54)_  = 1.373, *p* = .262). An interaction between gender and polymorphism was also not significant (*F*_(2, 51)_  = .596, *p* = .555). However, a three-way significant interaction emerged between gender, polymorphism and a SOA (*F*_(4, 102)_  = 3.279, *p*<.014). With the shortest SOA ( = 23 ms) female L/L homozygotes had higher rate of correct discrimination than S/L heterozygotes (*t*_(24)_  = −2.306, *p*<.030, Cohen's d = 1.19) while in males this pattern of results was reversed, although not at a significant level (*t*_(10)_  = 1.475, *p* = .171) (see Figure 6). If we consider two-alternative target discrimination in metacontrast as a task involving decision making under ambiguity (and especially so with shortest target-to-mask delay) then it follows from our results that female L/L homozygotes seem to manage this situation better than S/L heterozygotes, but males do not show this regularity. This is consistent with recent data showing a similar gender and 5-HTTLPR polymorphism interaction in decision making under ambiguity in a gambling task [24].

## Discussion

We found no overall effect of BDNF Val/Met, NRG1-rs6994992 or 5HTTLPR on visual discrimination in metacontrast masking, but a genotype x gender interaction was found with all three common gene variants examined. We should bear in mind that due to the exploratory format of the present study, including many variables and their combinations even the significant effects of interaction should be interpreted cautiously. We have not solved the problem of multiple comparisons and for this, more restricted and targeted future studies are necessary. However, on the other hand, our present results have made it possible to know better where the possible more rigorous effects could be found if specifically studied. It appears that when discrimination of target shapes under metacontrast masking has to be executed and the conditions are relatively more difficult, male subjects carrying alleles that are considered as less advantageous in terms of development of brain tissues seem to cope better with these situations compared to female subjects. The beginning stages of the experiment, intermediate or short SOAs instead of the long SOAs and mutually incongruent target/mask shapes can be considered as the more difficult conditions. Yet we see that male met allele carriers (Val66Met polymorphism), T allele carriers (NGR1) and S allele carriers (5HTTLPR) perform relatively better. On the other hand, the results we obtained with only three gene polymorphisms as variables in metacontrast already seem to show a high level of complexity and interactivity of different hereditary factors involved in visual masking and possibly other fast-perception phenomena. If we consider that in virtually all studies of masking carried out so far, (i) genetic factors have not been controlled and (ii) relatively small groups of subjects (e.g., from 4 to 10) have been used then – in the light of our present results – it should not be surprising how variable and often mutually inconsistent the results of masking research have been. This calls for further research where progressively more candidate genes as possible factors of gene expression ultimately manifesting in the variability of behavioural phenotypes of visual masking are tested. This also calls for more care in controlling the factors that interact with genetic effects such as gender, nationality, brain morphology, etc. Among the most difficult questions there will be the problem of whether gene polymorphisms are expressed directly in the brain mechanisms of basic vision or there is mainly an indirect influence mediated by higher cognitive and personality related processes that influence the ways subjects deal with perceptual representations formed in itself in an invariant species-specific way.
